# Rapid Evaluation of Wet Gluten Content in Wheat Using Hyperspectral Technology Combined with Machine Learning Algorithms

**DOI:** 10.3390/foods15010041

**Published:** 2025-12-23

**Authors:** Yan Lai, Yan-Yan Li, Min Sha, Peng Li, Zheng-Yong Zhang

**Affiliations:** 1 School of Management Science and Engineering, Nanjing University of Finance and Economics, Nanjing 210023, China; raiyeonn@gmail.com (Y.L.); minsha@nufe.edu.cn (M.S.); 2 Humanities and Social Sciences Laboratory of Jiangsu Province—Food Safety and National Strategic Governance, Jiangnan University, Wuxi 214122, China; 3 School of Food Science and Engineering, Nanjing University of Finance and Economics, Nanjing 210023, China; 1120220501@stu.nufe.edu.cn; 4 Key Laboratory of Food Processing and Quality Control, Nanjing University of Finance and Economics, Nanjing 210023, China

**Keywords:** hyperspectral, machine learning, chemometrics, content prediction

## Abstract

The development of rapid and intelligent methods is urgently needed for wheat quality evaluation. Using the prediction of wet gluten content as a case study, this work systematically investigated the performance of various machine learning algorithms and their optimization for content prediction, based on hyperspectral data from the visible and near-infrared ranges of wheat grains and flour. The results revealed that the random forest regression (RFR) algorithm delivered the best predictive performance under two conditions: first, when applied directly to visible spectra; and second, when applied to fused visible and near-infrared spectral data. This held true for both grains and flour. Conversely, its direct application to NIR spectra alone yielded relatively worse performance. Following data optimization, the first-derivative (FD) visible spectra of wheat grains were smoothed using a Savitzky–Golay (SG) filter and subsequently used as input for the RFR model. This optimized approach achieved a coefficient of determination (r^2^) of 0.8579, a root mean square error (RMSE) of 0.0216, and a relative percent deviation (RPD) of 2.6978. Under the same conditions, for wheat flour, the corresponding values were 0.8383, 0.0231, and 2.5293, respectively. Similarly, for wheat flour, the RFR model was applied to the SG-filtered FD spectra derived from the fused visible and near-infrared data, yielding an r^2^ of 0.8474, an RMSE of 0.0224, and an RPD of 2.6034. Under the same conditions, wheat grains yielded an r^2^ of 0.8494, an RMSE of 0.0223, and an RPD of 2.6208. This efficient and rapid intelligent prediction scheme demonstrates considerable potential for the quality assessment and control of relevant food products.

## 1. Introduction

In the routine quality monitoring of wheat and similar foods, analysis often focuses on quantifying specific chemical components. For instance, the content of wet gluten and protein in wheat directly influences the properties and functionality of its derived products and is closely tied to economic value. Wet gluten, a unique protein composite in wheat consisting primarily of gliadin and glutenin, serves as a key quality indicator. Traditional methods for determining wet gluten content, such as manual washing and instrumental techniques, are not only time-consuming and labor-intensive but also suffer from variable accuracy due to external and human factors. Furthermore, these methods are destructive and require multi-step sample preparation and pretreatment [1,2,3].

Spectral detection technology, owing to its rapid characterization and low destructiveness, has emerged as a promising technique for evaluating food composition [4,5]. The recent rise of machine learning algorithms has further propelled the effective integration of these two technologies, establishing it as a key research direction for rapid quality component prediction [6]. For instance, Wang et al. achieved rapid detection of rice protein content by fusing Raman and near-infrared spectroscopy, combined with an improved binary particle swarm optimization algorithm and partial least squares (PLS) [7]. Fan et al. employed near-infrared spectroscopy coupled with PLS regression, support vector machine (SVM), and extreme learning machine (ELM) models to quantitatively analyze protein content in different varieties of maize flour [8]. Peng et al. used terahertz time-domain spectroscopy combined with the sparrow algorithm, optimized SVM, and other algorithms to effectively identify wheat gluten types [9]. Compared to conventional spectroscopy, hyperspectral imaging (HSI) has recently gained widespread attention due to its non-destructive nature, broad spectral range, and imaging capability. It has become a vital data source for characterizing and predicting food quality attributes, with its integration with machine learning becoming increasingly imminent. For example, Cui et al. evaluated near-infrared HSI combined with a convolutional neural network (CNN) for rapid, non-destructive quality testing of fourteen wheat varieties in Ningxia, demonstrating excellent predictive performance [10]. Nargesi et al. utilized visible near-infrared HSI alongside artificial neural networks (ANNs) and PLS regression to estimate ash content in wheat flour, showing that this method provides accurate, rapid, and non-destructive estimation [11]. Olakanmi et al. employed HSI with PLS to classify and predict fava bean-fortified bread based on protein content [12]. Zhang et al. studied the prediction of protein and wet gluten content in wheat flour using hyperspectral and RGB sensors. By combining original reflectance, wavelet features, and color indices with the RFR algorithm, they achieved optimal accuracies of 0.864 and 0.847, respectively [13].

Nevertheless, more research is needed to address several unresolved questions. For instance, do hyperspectral visible, near-infrared, or their fusion modes yield comparable effects in machine learning-based content prediction? Furthermore, does the physical state (e.g., whole grain vs. flour) of the test sample significantly influence the performance of combined hyperspectral and machine learning algorithms?

This work collected visible and near-infrared hyperspectral data from wheat grains and flour with varying wet gluten contents, thereby expanding the range of sample states examined compared to prior studies. We systematically evaluated the predictive performance of different spectral modes—including single visible, single near-infrared, direct fusion, and feature fusion—using various machine learning algorithms. Furthermore, multiple preprocessing methods, feature extraction techniques, and their combinations were investigated to optimize the RFR algorithm, leading to further improvements in prediction accuracy. The findings provide technical support for the rapid identification and quality control of wheat.

## 2. Materials and Methods

### 2.1. Samples and Equipment

A total of 100 wheat samples with varying gluten contents were obtained from Jiangsu Jindudi Seed Industry Co., Ltd. (Yangzhou, China). The main wheat varieties come from Jiangsu and surrounding wheat-producing areas in China, including varieties such as Zhenmai 18 and Yangmai 31. Harvesting was performed during 2024, and wheat samples were stored in a 4 °C refrigerated environment after purchase. Hyperspectral images were acquired using a push-broom hyperspectral imaging system (Model: IRCP0078-ICOMB, manufactured by Wuling Optical Instrument Co., Ltd., Taiwan, China).

### 2.2. Determination of Wet Gluten Content

A 10 g sample of wheat flour was placed into the washing cup of a gluten analyzer, which was fitted with a polyester sieve (mesh size: 88 μm) at the bottom. The cup was gently shaken to form a smooth and even flour layer. Subsequently, 4.8 mL of a sodium chloride solution was added. The mixture was kneaded for 20 s and then washed for 5 min. During washing, 250–280 mL of sodium chloride washing solution was used at a controlled flow rate of 50–56 mL/min. After washing, the wet gluten was removed from the cup, gently pressed onto the sieve box of a centrifuge, and centrifuged at 6000 r/min for 1 min. The wet gluten was then immediately removed with metal tweezers, weighed, and its content was calculated.

### 2.3. Hyperspectral Data Acquisition

Visible and near-infrared hyperspectral images were acquired in reflectance mode using a line-scanning imaging system. The system comprised two cameras: an ImSpector V10E spectrograph (Wuling Optical Instrument Co., Ltd., Taiwan, China) covering the visible range (400–1000 nm, 558 spectral bands) and an ImSpector N17E spectrograph (Wuling Optical Instrument Co., Ltd., Taiwan, China) covering the near-infrared range (900–1700 nm, 483 spectral bands). Two halogen lamps, positioned approximately 350 mm from the translation stage, illuminated the samples at an optimal incident angle of about 45° relative to the detector. The scanning interval was set at 150 mm with a step distance of 100 mm. For imaging in the 400–1000 nm range, wheat grain and flour samples were placed in culture dishes (60 mm and 35 mm in diameter, respectively) on the stage. Using the spectrometer’s Spectral Image software(Wuling Optical Instrument Co., Ltd., Taiwan, China), the stage speed was set to 6.93 mm/s with exposure times of 15 ms for grains and 9 ms for flour. For the 900–1700 nm range, the stage speed was adjusted to 1.95 mm/s, with corresponding exposure times of 15 ms for grains and 11 ms for flour.

### 2.4. Dataset Description

The experimental dataset comprised 100 wheat samples with wet gluten concentrations ranging from 0.0662 to 0.4102. Specifically, 27, 45, and 28 samples fell within the concentration intervals of 0.3010–0.4102, 0.2427–0.2980, and 0.0662–0.2397, respectively (detailed distribution is provided in Table 1). For each sample in both wheat grain and flour states, hyperspectral data were acquired across two spectral ranges: the visible (400–1000 nm) and the near-infrared (900–1700 nm) regions.

### 2.5. Data Processing

The stability and predictive ability of the established models were evaluated using three metrics: the coefficient of determination (r^2^), the root mean square error (RMSE), and the relative percent deviation (RPD). A higher r^2^ value indicates a stronger linear correlation in the model. A lower RMSE corresponds to a smaller error between measured and predicted values. The RPD, defined as the ratio of the standard deviation (SD) to the standard error of prediction (SEP), reflects the model’s predictive capacity.

All machine learning and preprocessing algorithms involved in this study were implemented using MATLAB R2024a (MathWorks, Natick, MA, USA) on a personal computing platform. The algorithms included the following:

Machine learning models: partial least squares regression (PLSR), principal component regression (PCR), support vector regression (SVR), extreme learning machine (ELM), convolutional neural network (CNN), bidirectional long short-term memory network (BiLSTM), and random forest regression (RFR).

Spectral preprocessing & feature selection: normalization (NL), standard normal Variate (SNV), multiplicative scatter correction (MSC), Savitzky–Golay (SG) filtering, wavelet denoising (WD), first and second derivatives (FD, SD), competitive adaptive reweighted sampling (CARS), successive projections algorithm (SPA), and uninformative variable elimination (UVE).

The hardware consisted of a laptop equipped with a 13th Gen Intel^®^ Core™ i7-1360P processor (2.20 GHz) and 16.0 GB of RAM (Lenovo Co., Ltd., Beijing, China).

### 2.6. Algorithm Parameter Settings

The key parameters for each machine learning algorithm were set as follows:

When selecting principal components for principal component analysis (PCA), the primary criterion is that the cumulative contribution rate must exceed 95%. Separately, the PLSR and PCR algorithms determine the final number of components based on the minimum mean squared error of prediction (MSEP) using 10-fold cross-validation.

SVR: A Gaussian kernel was employed. Parameters were optimized via grid search coupled with 5-fold cross-validation to minimize the mean squared error (MSE). For the near-infrared spectra of wheat flour, the regularization parameter *C* and kernel parameter gamma (γ) were set to 1 and 0.5, respectively.

ELMR: The model was optimized through 5-fold cross-validation to minimize MSE. For wheat flour NIR data, the network structure comprised a single hidden layer with 500 nodes and a sine activation function.

CNNR: Training aimed to minimize the RMSE. The configuration was: Adam optimizer; maximum epochs: 100; initial learning rate: 0.001; batch size: 32; and L2 regularization factor: 0.001.

BiLSTMR: Training also targeted RMSE minimization. Using wheat flour NIR data (483 spectral points as sequential input) as an example, the architecture included a two-layer bidirectional LSTM (with 50 and 25 hidden units, respectively), three dropout layers, and a fully connected output layer of 10 nodes.

RFR: The number of trees was determined as 100 by observing the point where the out-of-bag error stabilized (below 0.001). The minimum number of samples required at a leaf node was set to 3 to control tree growth.

## 3. Results

### 3.1. Hyperspectral Characterization of Wheat

**Figure 1:**
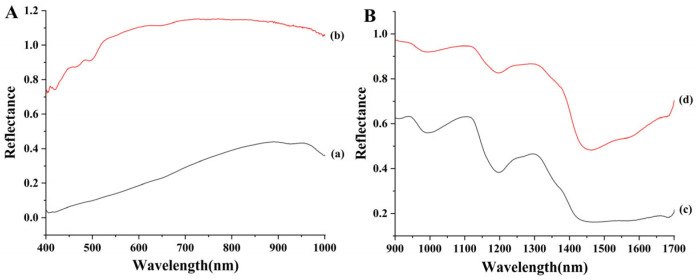
Hyperspectral images of wheat grain (a) and flour (b) in the visible range (400–1000 nm) (**A**), and of wheat grain (c) and flour (d) in the near-infrared range (900–1700 nm) (**B**).

Hyperspectral data were collected separately for wheat grains and flour, as presented in Figure 1. Figure 1A displays the visible spectral range (400–1000 nm). The absorption valley near 450 nm is associated with color characteristics, while the valley near 930 nm may correspond to the third overtone of C–H bonds in proteins [14]. Furthermore, the visible spectral reflectance of wheat grains is consistently lower than that of their flour counterpart from the same sample. This difference is likely attributable to a substantial increase in surface area after grinding. Figure 1B shows the near-infrared spectral range (900–1700 nm). The prominent peaks between 970 and 1380 nm are primarily related to the second overtones of N–H and C–H/C–H_2_ stretching vibrations. Key absorption peaks and valleys are observed around 990, 1120, 1195, 1300, 1450, and 1660 nm, which are mainly attributed to the doubling and combination bands arising from stretching vibrations of C–H or O–H groups [15,16]. Consistent with the visible range, the NIR reflectance of grains is also lower than that of flour, presumably for the same reason.

### 3.2. Prediction Based on Visible Spectra Combined with Machine Learning

Hyperspectral data from the visible range were first analyzed using a partial least squares regression (PLSR) model [17]. The dataset was randomly split into a training set (70%) and a test set (30%). To ensure reproducibility, a fixed random seed was used for this split. A *t*-test confirmed no significant difference in wet gluten content between the two sets (*p* = 0.843), validating the split’s representativeness. Given the high dimensionality (558 spectral variables) relative to the sample size, PCA was applied to mitigate overfitting risk. For wheat grain data, the first three principal components (cumulative variance explained: 99.83%) were selected based on minimizing the MSEP. The PLSR model built on these components yielded test set performance metrics: r^2^ = 0.7425, RMSE = 0.0291, and RPD = 2.0045. For wheat flour, the first three principal components (cumulative variance: 99.70%) were similarly selected, resulting in superior performance (r^2^ = 0.7960, RMSE = 0.0259, RPD = 2.2517). According to established interpretation [18], an RPD > 2 indicates good predictive ability. Therefore, the PLSR models, particularly for flour, demonstrate satisfactory predictive performance for wet gluten content based on visible spectra.

Principal component regression (PCR) was also employed for predictive analysis. For wheat grains, the first two principal components (PCs), explaining 99.04% of the variance, were selected. Although PCs result from mathematical transformation and cannot be uniquely assigned to specific spectral bands, analyzing their loadings reveals key contributing wavelengths. For PC1, these were clustered around 730–742 nm; for PC2, around 995–1000 nm. The resulting PCR model achieved a test set r^2^ of 0.7437, RMSE of 0.0291, and RPD of 2.0089. Similarly, for wheat flour, the first two PCs (98.39% variance explained) were used. The major contributing wavelengths were around 992–1000 nm for PC1 and 402–406 nm for PC2. The model performance parameters were r^2^ = 0.7938, RMSE = 0.0261, and RPD = 2.2398. Both models demonstrated reasonable predictive capability.

The predictive performance of SVR [19], ELMR [20], CNNR [21], BiLSTMNR [22], and RFR [23] algorithms was further evaluated using both the full visible spectrum data and the PCA-reduced data (Table 2, Table 3, Table 4 and Table 5). A comparison reveals distinct trends regarding PCA preprocessing. The prediction performance of SVR and BiLSTMR algorithms improved with PCA, whereas that of ELMR, CNNR, and RFR decreased. Among the traditional linear models, PLSR performed better on flour samples, while PCR showed a slight edge on grain samples. Notably, the RFR algorithm achieved the highest overall performance when applied directly to the full visible spectrum data (without PCA). For wheat grains, it yielded an r^2^ of 0.8261, an RMSE of 0.0239, and an RPD of 2.4393. For wheat flour, performance was marginally higher (r^2^ = 0.8331, RMSE = 0.0235, RPD = 2.4894). Bootstrap confidence intervals (95%) for the RFR model’s RMSE and r^2^ were [0.0134, 0.0332] and [0.7106, 0.9255], respectively. The relatively narrow intervals indicate stable and reliable model predictions.

### 3.3. Prediction Based on Near-Infrared Spectroscopy Combined with Machine Learning

Under the hyperspectral near-infrared mode, the sample data were similarly partitioned into training and test sets at a 7:3 ratio. The full near-infrared spectral data were then used in conjunction with various machine learning algorithms to predict wet gluten content. The corresponding evaluation metrics are summarized in Table 6 and Table 7. The results reveal considerable variation in predictive performance across different algorithms. The optimal performance was achieved by the RFR algorithm for wheat grains, yielding an r^2^ of 0.8012, an RMSE of 0.0256, and an RPD of 2.2812. Bootstrap confidence intervals (95%) for the RFR model’s RMSE and r^2^ were [0.0163, 0.0341] and [0.6738, 0.8975], respectively. These relatively narrow intervals indicate that the model’s predictions are stable and acceptable. For wheat flour, the best performance was observed with the ELMR algorithm, which produced an r^2^ of 0.7855, an RMSE of 0.0266, and an RPD of 2.1961, indicating a relatively robust predictive capability.

Subsequently, PCA was applied to the near-infrared spectral data (original dimensions: 483). The first three PCs accounted for 77.98%, 20.38%, and 1.39% of the total variance, respectively. Analysis of the PC loadings identified key wavelength regions: for wheat grains, PC1 was associated with wavelengths around 1185 and 1351–1358 nm, and PC2 with 901–908 nm. For wheat flour, PC1 was linked to 1691–1701 nm, and PC2, similarly, to 901–908 nm. These PCA-reduced features were then used as input for various machine learning algorithms (results in Table 8 and Table 9). Overall, predictive performance was generally poor across all models. The PLSR algorithm showed marginally better, yet still limited, performance for both grain and flour states. This outcome suggests that the relationship between the NIR spectral profiles (or their principal components) and gluten constituents may be less direct or more complex compared to that observed in the visible range, highlighting a modality-dependent difference in spectral information relevance.

### 3.4. Prediction Based on Fused Spectroscopy and Machine Learning

**Figure 2:**
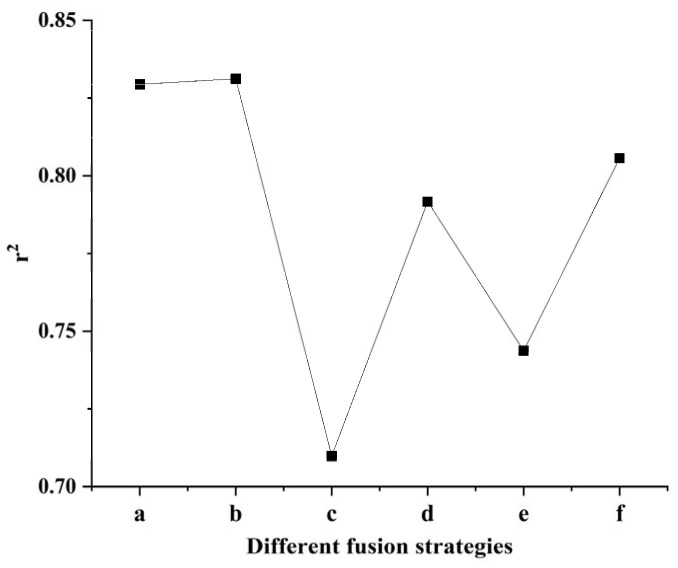
The r^2^ results of (a) RFR with direct spectral fusion data of wheat grains, (b) RFR with direct spectral fusion data of wheat flour, (c) ELMR with PCA feature extraction fusion data of wheat grains, (d) PLSR/PCR with PCA feature extraction fusion data of wheat flour, (e) PCR with PCA feature extraction fusion data of wheat grains and (f) PCR with PCA feature extraction fusion data of wheat flour.

To leverage comprehensive spectral information, visible and near-infrared data were integrated to predict wet gluten content using three distinct fusion strategies. (1) Direct spectral fusion: The raw visible and NIR spectra were directly concatenated and used as input for various machine learning algorithms. The results (Table 10 and Table 11) indicate that the RFR algorithm achieved the best prediction performance for both wheat grains and flour, with slightly superior results for flour. (2) Post-fusion PCA: The concatenated spectra were first fused and then subjected to PCA for dimensionality reduction. Subsequent model predictions (Table 12 and Table 13) were generally poor. For grains, the ELMR performed marginally better, while for flour, PLSR and PCR algorithms showed relatively better, though still limited, results. (3) Mode-wise PCA fusion: Visible and near-infrared spectra were independently reduced via PCA, and the extracted principal components from both modes were then merged for prediction (Table 14 and Table 15). Under this strategy, the principal component regression (PCR) algorithm yielded the best results for both sample states, again with flour models performing slightly better. A visual summary comparing the best r^2^ values achieved by various algorithms across all fusion methods is presented in Figure 2. In the figure, (a), (b) correspond to strategy 1 mentioned above; (c), (d) correspond to strategy 2; and (e), (f) correspond to strategy 3. The highest r^2^ was obtained by the RFR model using the direct fusion data on wheat flour. Bootstrap confidence intervals (95%) for the RFR model’s RMSE and r^2^ were [0.0144, 0.0329] and [0.7056, 0.9230], respectively. The relatively narrow intervals indicate stable and reliable model predictions.

### 3.5. Optimization of Prediction Based on Hyperspectral Data and Machine Learning

#### 3.5.1. Algorithm Performance Analysis and Selection of RFR

The preceding analyses identified the RFR algorithm as delivering superior predictive performance. This superiority can be attributed to several inherent advantages of RFR pertinent to our dataset: (1) Its ensemble nature, combining multiple decision trees, enhances stability and mitigates overfitting through bootstrap aggregation and random feature selection. (2) It effectively handles high-dimensional data and captures non-linear relationships and interactions within the spectral data. (3) Its training process, based on deterministic greedy partitioning, is more stable and less data-hungry compared to gradient-based optimization used by deep learning models.

In contrast, CNN/BiLSTM, which excels at extracting local spatial or sequential patterns, showed limited effectiveness in this study. Their high model complexity and reliance on large datasets for stable gradient optimization likely led to overfitting and higher variance, given our current sample size. Thus, RFR’s bias-variance trade-off and regularization mechanisms were better suited, yielding a decisive advantage.

#### 3.5.2. Optimization via Spectral Preprocessing and Feature Selection

Building on RFR’s strengths, we systematically explored optimization through spectral preprocessing and feature extraction [24,25,26,27]. Initial preprocessing of wheat grain visible spectra (NL, SNV, MSC, SG filtering, WD, FD, and SD) revealed that first-derivative (FD) processing yielded the greatest improvement (Table 16), achieving an r^2^ of 0.8451, RMSE of 0.0226, and RPD of 2.5843.

**Figure 3:**
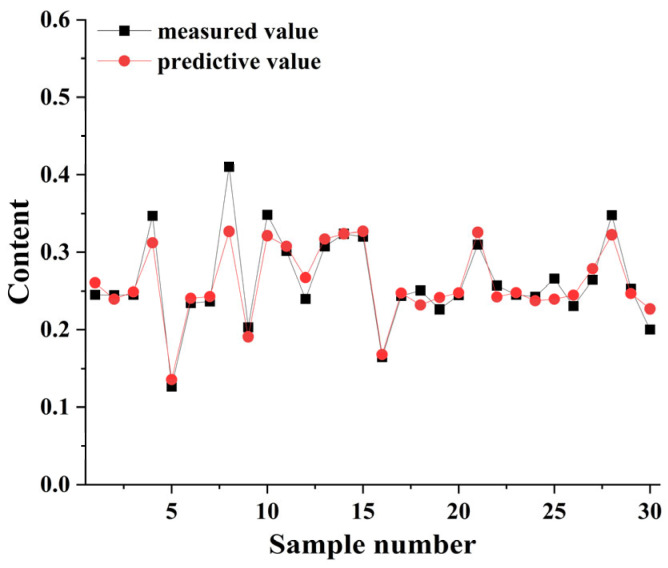
Predicted vs. measured wet gluten content for wheat grains using the RFR algorithm with FD and SG preprocessing.

Subsequent application of feature selection algorithms (CARS, SPA, and UVE) alone did not significantly enhance performance (Table 17). However, combining FD preprocessing with other techniques proved effective. The optimal combination was FD followed by Savitzky-Golay (SG) smoothing (Table 18), which produced the highest performance metrics: r^2^ = 0.8579, RMSE = 0.0216, RPD = 2.6978. The predicted vs. measured values for this model are shown in Figure 3, demonstrating close agreement. Incorporating UVE feature extraction after FD and SG processing (r^2^ = 0.8552, RMSE = 0.0218, RPD = 2.6732) was slightly less effective than the FD-SG combination alone.

Further optimization of the RFR algorithm was investigated under three conditions: fused visible and near-infrared spectra for grains, visible spectra for flour, and fused visible and near-infrared spectra for flour. For wheat grains with fused visible and near-infrared spectra, FD preprocessing yielded the most significant improvement (Table S1), achieving an r^2^ of 0.8579, an RMSE of 0.0216, and an RPD of 2.6978. Feature extraction methods alone led to performance degradation (Table S2). Combining FD with other techniques did not surpass the standalone FD effect; for instance, the FD-SG-UVE combination resulted in an r^2^ of 0.8424, which was still lower (Table S3). For wheat flour with visible spectra, SNV preprocessing provided the best enhancement (r^2^ = 0.8484, RMSE = 0.0224, RPD = 2.6122; Table S4). SPA feature extraction offered a minor improvement (Table S5). The optimal combination, FD-SG, achieved an r^2^ of 0.8383 (Table S6), yet still underperformed relative to SNV alone. For wheat flour with fused visible and near-infrared spectra, FD preprocessing again proved most effective (r^2^ = 0.8399, RMSE = 0.0230, RPD = 2.5420; Table S7). UVE feature extraction provided a slight gain (Table S8). The best combined method, FD-SG, reached an r^2^ of 0.8474 (Table S9), outperforming the FD-SG-UVE combination and all other tested strategies in this condition.

### 3.6. Further Discussion on Applicability and Limitations

This study demonstrates that RFR is an effective tool for predicting wet gluten content from wheat hyperspectral datasets. The methodological framework established here possesses considerable generalizability. It could potentially be extended to analyze other grains (e.g., for predicting protein or moisture in corn and rice), feedstuffs, and even other spectroscopic data types with limited samples, such as Raman or fluorescence spectroscopy. Furthermore, the model exhibited a degree of generalizability when predicting samples outside the immediate range used for modeling. For practical application, such as in industrial real-time quality control, the trained RFR model could be embedded into the hardware of portable or online spectrometers, enabling rapid prediction at the scale of seconds or even milliseconds.

Several limitations of this work should be acknowledged. First, the study was conducted on a dataset of 100 wheat samples. While this sample size is sufficient to demonstrate the advantage of RFR in small-sample scenarios, it may limit the model’s capacity to learn more extensive and complex spectral-gluten relationships. Second, all spectral data were acquired using a single hyperspectral imaging system under controlled laboratory conditions. Model performance may degrade significantly when transferred across different instruments or laboratory environments due to inherent inter-system variability. Finally, although RFR incorporates inherent mechanisms (e.g., ensemble learning and random feature selection) to mitigate overfitting, the risk is not entirely eliminated when working with a limited number of samples.

## 4. Conclusions

This study demonstrates that the RFR algorithm provides the best predictive performance for wheat wet gluten content using hyperspectral data (visible and near-infrared). For wheat grains, optimal results (r^2^ = 0.8579, RMSE = 0.0216, RPD = 2.6978) were achieved with visible spectra preprocessed by FD and SG smoothing. For wheat flour, the best performance (r^2^ = 0.8474, RMSE = 0.0224, RPD = 2.6034) was obtained using fused visible-near-infrared spectra processed with the same FD-SG method. This work establishes a rapid, intelligent hyperspectral prediction method for wet gluten. Future research should focus on (1) the simultaneous prediction of multiple quality components, and (2) monitoring dynamic changes in these components during storage.

## Figures and Tables

**Table 1:** Concentration ranges and sample counts for wet gluten in wheat.

Number of Samples	Minimum Value	Maximum Value	Mean Value	Standard Deviation
27	0.3010	0.4102	0.3302	0.0257
45	0.2427	0.2980	0.2574	0.0152
28	0.0662	0.2397	0.1997	0.0526

**Table 2:** Performance of machine learning algorithms using the full visible spectrum data of wheat grains.

	PLSR	PCR	SVR	ELMR	CNNR	BiLSTMR	RFR
r^2^	-	-	−0.0994	0.7580	0.6747	−0.4317	**0.8261**
RMSE	-	-	0.0602	0.0282	0.0328	0.0687	**0.0239**
RPD	-	-	0.9700	2.0675	1.7833	0.8500	**2.4393**

Note: “-” indicates no calculation result.

**Table 3:** Performance of machine learning algorithms using the full visible spectrum data of wheat flour.

	PLSR	PCR	SVR	ELMR	CNNR	BiLSTMR	RFR
r^2^	-	-	0.3614	0.7926	0.5065	−0.1778	**0.8331**
RMSE	-	-	0.0459	0.0262	0.0403	0.0623	**0.0235**
RPD	-	-	1.2727	2.2333	1.4478	0.9372	**2.4894**

Note: “-” indicates no calculation result.

**Table 4:** Performance of machine learning algorithms using PCA-processed visible spectral data of wheat grains.

	PLSR	PCR	SVR	ELMR	CNNR	BiLSTMR	RFR
r^2^	0.7425	**0.7437**	−0.1045	0.4836	0.5570	0.4853	0.7139
RMSE	0.0291	**0.0291**	0.0603	0.0413	0.0382	0.0412	0.0307
RPD	2.0045	**2.0089**	0.9678	1.4154	1.5282	1.4177	1.9015

**Table 5:** Performance of machine learning algorithms using PCA-processed visible spectral data of wheat flour.

	PLSR	PCR	SVR	ELMR	CNNR	BiLSTMR	RFR
r^2^	**0.7960**	0.7938	0.4749	0.6299	0.5254	0.2801	0.7577
RMSE	**0.0259**	0.0261	0.0416	0.0349	0.0396	0.0487	0.0283
RPD	**2.2517**	2.2398	1.4035	1.6719	1.4764	1.1987	2.0664

**Table 6:** Performance of machine learning algorithms using the full near-infrared spectrum of wheat grains.

	PLSR	PCR	SVR	ELMR	CNNR	BiLSTMR	RFR
r^2^	-	-	0.3153	0.6962	0.4382	−0.6179	**0.8012**
RMSE	-	-	0.0475	0.0316	0.0430	0.0730	**0.0256**
RPD	-	-	1.2292	1.8454	1.3570	0.7996	**2.2812**

Note: “-” indicates no calculation result.

**Table 7:** Performance of machine learning algorithms using the full near-infrared spectrum of wheat flour.

	PLSR	PCR	SVR	ELMR	CNNR	BiLSTMR	RFR
r^2^	-	-	0.1952	**0.7855**	0.4393	−0.4068	0.6417
RMSE	-	-	0.0515	**0.0266**	0.0430	0.0681	0.0344
RPD	-	-	1.1338	**2.1961**	1.3583	0.8575	1.6992

Note: “-” indicates no calculation result.

**Table 8:** Performance of machine learning algorithms using PCA-processed near-infrared spectral data of wheat grains.

	PLSR	PCR	SVR	ELMR	CNNR	BiLSTMR	RFR
r^2^	**0.5272**	0.3524	0.3117	0.4261	0.1183	0.1998	0.3978
RMSE	**0.0395**	0.0462	0.0476	0.0435	0.0539	0.0514	0.0446
RPD	**1.4792**	1.2639	1.2260	1.3426	1.0832	1.1370	1.3106

**Table 9:** Performance of machine learning algorithms using PCA-processed near-infrared spectral data of wheat flour.

	PLSR	PCR	SVR	ELMR	CNNR	BiLSTMR	RFR
r^2^	**0.6044**	0.3942	0.1597	0.1185	0.1729	−0.0120	0.4914
RMSE	**0.0361**	0.0447	0.0526	0.0539	0.0522	0.0578	0.0410
RPD	**1.6171**	1.3067	1.1095	1.0833	1.1184	1.0110	1.4261

**Table 10:** Performance of machine learning algorithms using direct spectral fusion data of wheat grains.

	PLSR	PCR	SVR	ELMR	CNNR	BiLSTMR	RFR
r^2^	-	-	0.1242	0.6954	0.6835	−0.3927	**0.8294**
RMSE	-	-	0.0537	0.0317	0.0323	0.0678	**0.0237**
RPD	-	-	1.0868	1.8430	1.8080	0.8618	**2.4628**

Note: “-” indicates no calculation result.

**Table 11:** Performance of machine learning algorithms using direct spectral fusion data of wheat flour.

	PLSR	PCR	SVR	ELMR	CNNR	BiLSTMR	RFR
r^2^	-	-	0.2819	0.5665	0.6849	−0.3705	**0.8312**
RMSE	-	-	0.0487	0.0378	0.0322	0.0672	**0.0236**
RPD	-	-	1.2002	1.5448	1.8119	0.8688	**2.4759**

Note: “-” indicates no calculation result.

**Table 12:** Performance of machine learning algorithms using post-fusion PCA spectral features of wheat grains.

	PLSR	PCR	SVR	ELMR	CNNR	BiLSTMR	RFR
r^2^	0.5744	0.5713	0.1213	**0.7098**	0.5350	0.4944	0.5599
RMSE	0.0375	0.0376	0.0538	**0.0309**	0.0392	0.0408	0.0381
RPD	1.5591	1.5535	1.0851	**1.8879**	1.4915	1.4304	1.5331

**Table 13:** Performance of machine learning algorithms using post-fusion PCA spectral features of wheat flour.

	PLSR	PCR	SVR	ELMR	CNNR	BiLSTMR	RFR
r^2^	**0.7917**	**0.7917**	0.3856	0.6965	0.5137	0.2970	0.7839
RMSE	**0.0262**	**0.0262**	0.0450	0.0316	0.0400	0.0481	0.0267
RPD	**2.2284**	**2.2284**	1.2976	1.8462	1.4585	1.2131	2.1881

**Table 14:** Performance of machine learning algorithms using mode-wise PCA fusion spectral features of wheat grains.

	PLSR	PCR	SVR	ELMR	CNNR	BiLSTMR	RFR
r^2^	0.7187	**0.7437**	0.1241	0.6740	0.3729	0.4831	0.7149
RMSE	0.0305	**0.0291**	0.0537	0.0328	0.0455	0.0413	0.0307
RPD	1.9177	**2.0089**	1.0868	1.7813	1.2843	1.4147	1.9049

**Table 15:** Performance of machine learning algorithms using mode-wise PCA fusion spectral features of wheat flour.

	PLSR	PCR	SVR	ELMR	CNNR	BiLSTMR	RFR
r^2^	0.6515	**0.8057**	0.3502	0.6652	0.4631	0.1313	0.6610
RMSE	0.0344	**0.0253**	0.0463	0.0332	0.0421	0.0535	0.0334
RPD	1.7061	**2.3076**	1.2617	1.7577	1.3881	1.0913	1.7469

**Table 16:** Performance of the RFR algorithm with different preprocessing methods on the full visible spectrum of wheat grains.

	Raw	NL	SNV	MSC	SG	WD	FD	SD
r^2^	0.8261	0.8261	0.6299	0.6419	0.8307	0.8181	**0.8451**	0.7294
RMSE	0.0239	0.0239	0.0349	0.0344	0.0236	0.0245	**0.0226**	0.0299
RPD	2.4393	2.4393	1.6719	1.6997	2.4722	2.3847	**2.5843**	1.9551

**Table 17:** Performance of the RFR algorithm with different feature extraction methods on the full visible spectrum of wheat grains.

	Raw	PCA	CARS	SPA	UVE
r^2^	**0.8261**	0.7139	0.7013	0.7600	0.7799
RMSE	**0.0239**	0.0307	0.0314	0.0281	0.0269
RPD	**2.4393**	1.9015	1.8611	2.0761	2.1679

**Table 18:** Performance of the RFR algorithm with different fused processing strategies on the full visible spectrum of wheat grains.

	Raw	FD	FD + NL	FD + SNV	FD + MSC	FD + SG	FD + WD	FD + UVE
r^2^	0.8261	0.8451	0.8451	0.6955	0.6961	**0.8579**	0.6135	0.8373
RMSE	0.0239	0.0226	0.0226	0.0317	0.0317	**0.0216**	0.0357	0.0232
RPD	2.4393	2.5843	2.5843	1.8433	1.8451	**2.6978**	1.6360	2.5212

## Data Availability

The original contributions presented in the study are included in the article/Supplementary Materials, further inquiries can be directed to the corresponding authors.

## Supplementary Materials

The following supporting information can be downloaded at: https://www.mdpi.com/article/10.3390/foods15010041/s1, Table S1: Performance of the RFR algorithm with different preprocessing methods on direct spectral fusion data of wheat grains. Table S2: Performance of the RFR algorithm with different feature extraction methods on direct spectral fusion data of wheat grains. Table S3: Performance of the RFR algorithm with different fused processing strategies on direct spectral fusion data of wheat grains. Table S4: Performance of the RFR algorithm with different preprocessing methods on the full visible spectrum of wheat flour. Table S5: Performance of the RFR algorithm with different feature extraction methods on the full visible spectrum of wheat flour. Table S6: Performance of the RFR algorithm with different fused processing strategies on the full visible spectrum of wheat flour. Table S7: Performance of the RFR algorithm with different preprocessing methods on direct spectral fusion data of wheat flour. Table S8: Performance of the RFR algorithm with different feature extraction methods on direct spectral fusion data of wheat flour. Table S9: Performance of the RFR algorithm with different fused processing strategies on direct spectral fusion data of wheat flour.
