# Supplementary material for: Rapid Evaluation of Wet Gluten Content in Wheat Using Hyperspectral Technology Combined with Machine Learning Algorithms
Source: Foods. 2025 Dec 23;15(1):41. doi: 10.3390/foods15010041 (PMC12785360; doi:10.3390/foods15010041)
Supplement: Supplementary file 1 [file foods-15-00041-s001.zip › foods-4028114-supplementary.pdf]

Supplementary Materials

Rapid evaluation of wet gluten content in wheat using hyperspectral technology combined with machine learning algorithms

Yan Lai[1], Yan-Yan Li[3], Min Sha[1,4], Peng Li[3*], Zheng-Yong Zhang[1,2,4*]

[1] School of Management Science and Engineering, Nanjing University of Finance and Economics, Nanjing 210023, Jiangsu, China; raiyeonn@gmail.com (Y. L.); 1120220501@stu.nufe.edu.cn (Y.-Y. L.); minsha@nufe.edu.cn (M. S.)

[2] Humanities and Social Sciences Laboratory of Jiangsu Province—Food Safety and National Strategic Governance, Jiangnan University, Wuxi 214122, Jiangsu, China

[3] School of Food Science and Engineering, Nanjing University of Finance and Economics, Nanjing 210023, Jiangsu, China

[4] Key Laboratory of Food Processing and Quality Control, Nanjing University of Finance and Economics, Nanjing 210023, Jiangsu, China

* Correspondence: zyzhang@nufe.edu.cn (Z.-Y. Z.); 9120151012@nufe.edu.cn (P. L.)

Table S1 Performance of the RFR algorithm with different preprocessing methods on direct spectral fusion data of wheat grains.

	Raw	NL	SNV	MSC	SG	WD	FD	SD
r^2	0.8294	0.8294	0.6016	0.6267	0.8215	0.8440	**0.8579**	0.8212
RMSE	0.0237	0.0237	0.0362	0.0351	0.0243	0.0227	**0.0216**	0.0243
RPD	2.4628	2.4628	1.6115	1.6647	2.4075	2.5755	**2.6978**	2.4051

Table S2 Performance of the RFR algorithm with different feature extraction methods on direct spectral fusion data of wheat grains.

	Raw	PCA	CARS	SPA	UVE
r^2	**0.8294**	0.5599	0.8088	0.7882	0.8206
RMSE	**0.0237**	0.0381	0.0251	0.0264	0.0243
RPD	**2.4628**	1.5331	2.3259	2.2098	2.4016

Table S3 Performance of the RFR algorithm with different fused processing strategies on direct spectral fusion data of wheat grains.

	Raw	FD	FD+NL	FD+SNV	FD+MSC	FD+SG	FD+WD	FD+UVE
r^2	0.8294	**0.8579**	0.8579	0.8333	0.8243	0.8494	0.6967	0.8268
RMSE	0.0237	**0.0216**	0.0216	0.0234	0.0241	0.0223	0.0316	0.0239
RPD	2.4628	**2.6978**	2.6978	2.4908	2.4265	2.6208	1.8467	2.4438

Table S4 Performance of the RFR algorithm with different preprocessing methods on the full visible spectrum of wheat flour.

	Raw	NL	SNV	MSC	SG	WD	FD	SD
r^2	0.8331	0.8331	**0.8484**	0.8270	0.8306	0.8397	0.8283	0.7967
RMSE	0.0235	0.0235	**0.0224**	0.0239	0.0236	0.0230	0.0238	0.0259
RPD	2.4894	2.4894	**2.6122**	2.4451	2.4710	2.5401	2.4549	2.2557

Table S5 Performance of the RFR algorithm with different feature extraction methods on the full visible spectrum of wheat flour.

	Raw	PCA	CARS	SPA	UVE
r^2	0.8331	0.7577	0.8213	**0.8354**	0.8273
RMSE	0.0235	0.0283	0.0243	**0.0233**	0.0239
RPD	2.4894	2.0664	2.4060	**2.5072**	2.4473

Table S6 Performance of the RFR algorithm with different fused processing strategies on the full visible spectrum of wheat flour.

	Raw	FD	FD+NL	FD+SNV	FD+MSC	FD+SG	FD+WD	FD+UVE
r^2	0.8331	0.8283	0.8283	0.8064	0.8019	**0.8383**	0.7534	0.8122
RMSE	0.0235	0.0238	0.0238	0.0253	0.0256	**0.0231**	0.0285	0.0249
RPD	2.4894	2.4549	2.4549	2.3115	2.2852	**2.5293**	2.0482	2.3467

Table S7 Performance of the RFR algorithm with different preprocessing methods on direct spectral fusion data of wheat flour.

	Raw	NL	SNV	MSC	SG	WD	FD	SD
r^2	0.8312	0.8312	0.8041	0.8122	0.8278	0.8219	**0.8399**	0.8247
RMSE	0.0236	0.0236	0.0254	0.0249	0.0238	0.0242	**0.0230**	0.0240
RPD	2.4759	2.4759	2.2978	2.3472	2.4510	2.4102	**2.5420**	2.4289

Table S8 Performance of the RFR algorithm with different feature extraction methods on direct spectral fusion data of wheat flour.

	Raw	PCA	CARS	SPA	UVE
r^2	0.8312	0.7839	0.8268	0.8145	**0.8344**
RMSE	0.0236	0.0267	0.0239	0.0247	**0.0234**
RPD	2.4759	2.1881	2.4437	2.3615	**2.4994**

Table S9 Performance of the RFR algorithm with different fused processing strategies on direct spectral fusion data of wheat flour.

	Raw	FD	FD+NL	FD+SNV	FD+MSC	FD+SG	FD+WD	FD+UVE
r^2	0.8312	0.8399	0.8399	0.8149	0.8014	**0.8474**	0.7376	0.8462
RMSE	0.0236	0.0230	0.0230	0.0247	0.0256	**0.0224**	0.0294	0.0225
RPD	2.4759	2.5420	2.5420	2.3640	2.2821	**2.6034**	1.9857	2.5936
